# Dopamine-Induced Ascorbate Release From Retinal Neurons Involves Glutamate Release, Activation of AMPA/Kainate Receptors and Downstream Signaling Pathways

**DOI:** 10.3389/fnins.2019.00453

**Published:** 2019-05-09

**Authors:** Camila Cabral Portugal, Thaísa Godinho da Encarnação, Ivan Domith, Alexandre dos Santos Rodrigues, Nádia Almeida de Oliveira, Renato Socodato, Roberto Paes-de-Carvalho

**Affiliations:** ^1^Instituto de Investigação e Inovação em Saúde and Instituto de Biologia Molecular e Celular (IBMC), Universidade do Porto, Porto, Portugal; ^2^Department of Neurobiology and Program of Neurosciences, Institute of Biology, Fluminense Federal University, Niterói, Brazil

**Keywords:** SVCT2, vitamin C, D-aspartate, excitatory amino acid transporters, D_1_R, EPAC, AKT, ERK

## Abstract

Ascorbate, the reduced form of Vitamin C, is one of the most abundant and important low-molecular weight antioxidants in living tissues. Most animals synthesize vitamin C, but some primates, including humans, have lost this capacity due to disruption in L-gulono-gamma-lactone oxidase gene. Because of this incapacity, those animals must obtain Vitamin C from the diet. Ascorbate is highly concentrated in the central nervous system (CNS), including the retina, and plays essential roles in neuronal physiology. Ascorbate transport into cells is controlled by Sodium Vitamin C Co-Transporters (SVCTs). There are four SVCT isoforms and SVCT2 is the major isoform controlling ascorbate transport in the CNS. Regarding ascorbate release from retinal neurons, Glutamate, by activating its ionotropic receptors leads to ascorbate release via the reversion of SVCT2. Moreover, dopamine, via activation of D_1_ receptor/cyclic AMP/EPAC2 pathway, also induces ascorbate release via SVCT2 reversion. Because the dopaminergic and glutamatergic systems are interconnected in the CNS, we hypothesized that dopamine could regulate ascorbate release indirectly, via the glutamatergic system. Here we reveal that dopamine increases the release of D-Aspartate from retinal neurons in a way independent on calcium ions and dependent on excitatory amino acid transporters. In addition, dopamine-dependent SVCT2 reversion leading to ascorbate release occurs by activation of AMPA/Kainate receptors and downstream ERK/AKT pathways. Overall, our data reveal a dopamine-to-glutamate signaling that regulates the bioavailability of ascorbate in neuronal cells.

## Introduction

In spite of not being an amine, ascorbate is also called Vitamin C as an extension of the term “vital amine” introduced by Casimir Funk in early twenties to indicate the nutritional factor necessary to prevent scurvy. At that time, the chemical identity of Vitamin C was not known. Later on, Albert Szent-Giörgyi identified a 6-carbon sugar obtained from acid fruits and adrenal glands, the “hexuronic acid” that was later termed ascorbic acid because of its anti-scurvy properties (Szent-Györgyi, 1963). Vitamin C is highly concentrated in the central nervous system (CNS), including the retina, where it is best known for its antioxidant properties. Besides, Vitamin C plays important roles in neural physiology, for instance, participating in the formation of the myelin shaft (Carey and Todd, 1987; Eldridge, 1987), regulating the release of acetylcholine (Kuo and Yoshida, 1980; Feuerstein et al., 1993), modulating NMDA receptor function (Majewska et al., 1990; Domith et al., 2018), modulating GABAergic neurotransmission (Calero et al., 2011) and acting as a co-factor in a plethora of enzymatic reactions such as the conversion of dopamine into norepinephrine (Rebec and Pierce, 1994) and the synthesis of neuropeptides (Glombotski et al., 1986). Regarding its oxidative states, Vitamin C can be found in two forms in living tissues, the oxidized form, dehydroascorbate (DHA), and the reduced form, ascorbate. DHA is taken up by glucose transporters (GLUT) 1, 2, 3 and 4 (Rumsey et al., 1997, 2000; Mardones et al., 2011) whereas ascorbate is taken up by the Sodium Vitamin C co-Transporter – SVCT (Slc23) (Daruwala et al., 1999; Tsukaguchi et al., 1999; Wang et al., 2000; Takanaga et al., 2004). There are two different SVCT isoforms, SVCT1 and 2 (Slc23a1 and Slc23a2). SVCT1 is found in epithelial tissues involved in ascorbate (re)absorption, while SVCT2 is highly expressed in the CNS. SVCT2 is a glycoprotein with 12 transmembrane domains that transports ascorbate in a sodium-dependent manner (Tsukaguchi et al., 1999), with potential N-glycosylation sites in the extracellular loop between transmembrane segments three and four (Tsukaguchi et al., 1999). This transporter can be regulated by multiple signaling pathways including PKA (Wu et al., 2007), PKC (Daruwala et al., 1999; Liang et al., 2002) and NO-cGMP-PKG-NF-kB (Portugal et al., 2012).

Although SVCTs are the major transporter systems regulating ascorbate uptake into cells, several mechanisms can be employed to mediate the release of ascorbate from cells (Corti et al., 2010). In one of such mechanisms, ascorbate can be released via the reversion of its high-affinity transporter SVCT2 (Portugal et al., 2009; da Encarnação et al., 2018). In neuronal cells, the neurotransmitters glutamate, acting on AMPA/kainate ionotropic receptors (Portugal et al., 2009), and dopamine (DA), acting through the D_1_R/cAMP/EPAC2 pathway (da Encarnação et al., 2018), can trigger SVCT2 reversion leading to ascorbate release. Because both glutamate and DA (two major neurotransmitter systems in the CNS) promote SVCT2-induced ascorbate release from neurons, we asked if these two neurotransmitter systems were, somehow, linked to mediate the release of ascorbate. Here we revealed that DA triggers D-aspartate release (a measure of glutamate release) through a calcium-independent and excitatory amino acid (EAA) transporter-dependent mechanism. Glutamate then activates AMPA/kainate receptors and downstream ERK and AKT pathways leading to SVCT2-dependent ascorbate release from neurons. Our data describe a DA-to-glutamate signaling regulating the bioavailability of ascorbate in the CNS.

## Materials and Methods

### Animals

Fertilized White Leghorn chicken eggs were obtained from a local hatchery and incubated at 38°C in a humidified atmosphere until the 8th day *in ovo*. All experiments were performed in compliance with ARRIVE guidelines and under institutional approval of COBEA (Ethical principles of animal experimentation) and the Committee on Ethics in Animal Research (CEPA) of the Universidade Federal Fluminense (number 00146/09).

### Reagents

Dopamine; SKF-38393 [1-phenyl-2,3,4,5-tetrahydro-(1H)-3-benzazepine-7,8-diol hydrochloride]; 8-pCPT-2′-O-Me-cAMP [8-(4-chlorophenylthio)-2′-O-methyladenosine 3′,5′-cyclic mon- ophosphate monosodium hydrate]; HEPES (4-(2-hydroxyethyl)- 1-piperazineethanesulfonic acid); DNQX (6,7-dinitroquinox- aline-2,3-dione); dimethyl sulfoxide; MK-801 (5S,10R)-(+)-5-Methyl-10,11-dihydro-5H-dibenzo[a, d]cyclohepten-5,10-imine hydrogen maleate); BAPTA-AM [1,2-bis-(o-Aminophenoxy)-ethane-N,N,-N′,N′-tetraacetic acid tetraacetoxy-Methyl ester]; PD 98,059 [2-(2-Amino-3-methoxyphenyl)-4H-1-benzopyran- 4-one]; UO126 [1,4-Diamino-2,3-dicyano-1,4-bis (o-amino- phenylmercapto) butadiene monoethanolate] and bovine serum albumin (BSA) were from Sigma Aldrich. Trypsin; minimum essential medium (MEM); glutamine; Penicillin; streptomycin and fetal bovine serum (FBS) were from Thermo Fisher Scientific. Acrylamide; ammonium persulphate (APS); N,N′-methylene-bisacrylamide; sodium dodecyl sulfate (SDS); tetramethyl-ethylenediamine (TEMED); ECL kit; polyvinylidene fluoride (PVDF) membranes; anti-mouse and anti-rabbit HRP-conjugated secondary antibodies were from GE Healthcare. DL-threo-β-benzyloxyaspartic acid (TBOA) was purchased from Tocris. 2-amino-5-phosphonopentanoic acid (APV) and LY294002 (2-(4-Morpholinyl)-8-phenyl-1(4H)-benzopyran-4-one hydrochloride) were purchased from Biomol. [^14^C] Ascorbic Acid (13 mCi/mmol) and [^3^H] D-aspartic acid (12.2 Ci/mmol) were from PerkinElmer. Primary antibodies against phospho-AKT (Ser473), AKT, phospho-ERK (Thr202/Tyr204) and ERK were from Cell Signaling.

### Primary Cultures of Retinal Cells

Monolayer cultures of chick retinal cells were prepared as previously described (de Mello, 1978). Briefly, retinas from 8-day-old chick embryos (E8) were dissected from other ocular tissues, including the retinal pigment epithelium, and digested with 0.2% trypsin in calcium and magnesium-free Hank’s balanced salt solution (HBSS), for 15 min at 37°C. Cells were then physically dissociated in MEM supplemented with 3% FBS, penicillin (100 U/mL), streptomycin (100 mg/mL), and glutamine (2 mM). After that, cultures were seeded (2 × 10^4^cells/mm^2^) in plastic dishes and maintained at 37°C in a humidified incubator with 95% air and 5% CO_2_. Culture medium was completely exchanged for fresh medium after 1 day in culture (C1) and experiments were performed at C3–C4. The proportion of neurons (80%) to Müller glia (20%) has been characterized elsewhere (Portugal et al., 2012).

### [^14^C] Vitamin C Release

Vitamin C release experiments were done exactly as previously described (Portugal et al., 2009). Firstly, the medium was removed and cultures were rinsed twice with HBSS (140 mM NaCl; 5 mM KCl; 20 mM HEPES; 4 mM glucose; 1 mM MgCl_2_, and 2 mM CaCl_2_, pH 7.4). After that, cultures were incubated with [^14^C] ascorbate (0.3 μCi/mL) for 40 min at 37°C and then rinsed twice with HBSS and further incubated with HBSS during four periods of 3 min to completely remove extracellular radioactivity. Cells were then incubated for 10 min with HBSS in order to estimate the [^14^C] ascorbate basal release and incubated for another period of 10 min with HBSS containing different antagonists. A third period of 10 min of incubation was performed with HBSS containing DA, SKF-38393, or Me-cAMP in the absence or in the presence of different antagonists. After all incubation periods, cells were then lysed with 5% trichloroacetic acid. All three supernatants sequentially collected from the same well, in addition to the cell lysates, were reserved to measure the radioactivity by liquid scintillation spectroscopy. Results were normalized to percent of control after calculation of the percent fractional release, that is, the percent of radioactivity released compared to intracellular radioactivity at each period of time. All Ascorbate release assays were conducted at 37^∘^C.

### [^3^H] D-Aspartate Release

Firstly, the medium was removed, the cultures rinsed twice with HBSS and then pre-incubated with [^3^H] D-Aspartate (1 μCi/mL) for 90 min. After this uptake period, the cultures were rinsed twice with HBSS and further washed for four periods of 3 min to completely remove the extracellular radioactivity. Cells were then incubated for 10 min with HBSS in order to measure the basal release. After this time, cultures were incubated for another period of 10 min with HBSS containing different drugs or antagonists. A third period of 10 min of incubation was performed with HBSS containing DA in the absence or presence of different antagonists. After this time, the cells were lysed with water and all three supernatants sequentially collected from the same well, in addition to the cell lysates, were reserved to measure the radioactivity by liquid scintillation spectroscopy. Results were normalized to percent of control after calculation of the percent fractional release, that is, the percent of radioactivity released compared to intracellular radioactivity at each period of time. All D-Aspartate release assays were conducted at 37^∘^C.

### Western Blotting

Cultures were washed twice with HBSS and starved for 40 min in HBSS to reduce basal phosphorylation of AKT and ERK. Afterward, cultures were incubated with DA, SKF-38393, or 8-pCPT-2′-O-Me-cAMP for different periods, washed twice with HBSS, lysed, and the total protein amount was estimated by Bradford’s method. Samples containing 60 μg protein were submitted to 9% SDS-PAGE and proteins transferred to PVDF membranes, which were then incubated overnight with specific antibodies against phosphorylated forms of ERK (1:1,000) or AKT (1:1,000). Subsequently, membranes were washed in TBS-T buffer, incubated with anti-rabbit HRP-conjugated secondary antibody (1:2,000) and developed using an ECL kit. After stripping with 0.2M glycine, pH 2.2, for 30 min, membranes were re-probed with anti-ERK and anti-AKT (1:1,000). Subsequently, membranes were washed in TBS-T and incubated with anti-rabbit (1:2,000) peroxidase-conjugated secondary antibody. Western blot quantifications were performed using the ImageJ software.

### Statistical Analysis

Data in all histograms display the mean ± SEM. Statistical analyses were performed using Student’s *t*-test or one-way ANOVA followed by the Bonferroni or Dunnet post-test using the GraphPad Prism Software.

## Results

We previously demonstrated that glutamate, by interacting with its ionotropic receptors, stimulates the release of ascorbate from cultured retinal cells (Portugal et al., 2009). The glutamate-induced ascorbate release is independent of calcium and mediated by the reversion of SVCT2 (Portugal et al., 2009). Moreover, by interacting with D_1_R and activating downstream EPAC2 pathway, DA induces ascorbate release also via SVCT2 reversion (da Encarnação et al., 2018). Because both the glutamatergic and the dopaminergic systems induce ascorbate release from neurons via the reversion of SVCT2, and the glutamatergic and dopaminergic systems are tightly associated in the retina, we studied whether the DA- and the glutamate-induced ascorbate release were, somehow, interconnected.

### Activation of D_1_R/EPAC2 Pathway by DA Drives Excitatory Amino Acid Release via Excitatory Amino Acid Transporter

**FIGURE 1:**
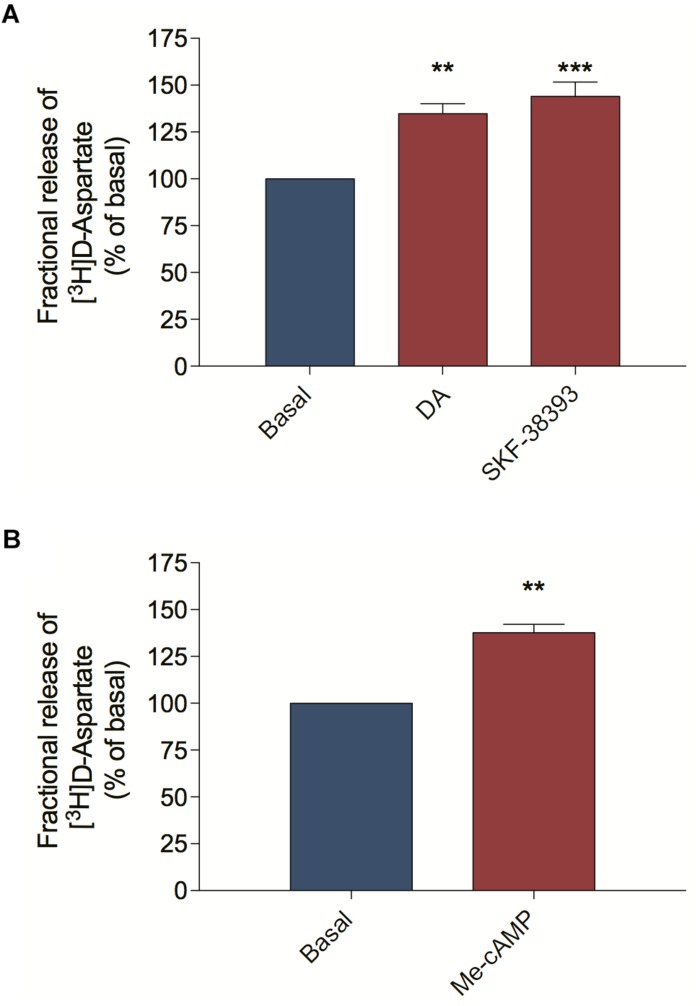
D_1_ receptor activation and EPAC stimulation trigger D-aspartate release from retinal cultures. Retinal cultures were incubated for 90 min with [^3^H] D-aspartate (1 μCi/mL), washed and processed for release experiments as described in section “Materials and Methods.” Cultures were incubated with DA (50 μM; **A**) or the D_1_ receptor agonist SKF-38393 (10 μM; **A**) or the selective EPAC activator Me-cAMP (100 μM; **B**) for 10 min. The results represent the mean ± SEM of three independent experiments. Statistical analyses were performed using one-way ANOVA followed by the Dunnet post-test **(A)** or the unpaired *t*-test **(B)**. **p < 0.01 (relative to basal); ***p < 0.001 (relative to basal). DA, Dopamine; Me-cAMP, 8-pCPT-2′-O-Me-cAMP.

Because the dopaminergic and the glutamatergic systems are interconnected in the retina and both systems induce ascorbate release by SVCT2 reversion, we asked if ascorbate release, elicited by DA might be mediated via EAA, including glutamate. To study EAA release from retinal cells we performed release assays using the non-metabolizable EAA [^3^H] D-Aspartate, which shares some transport mechanisms with endogenous glutamate (Palmer and Reiter, 1994; Muzzolini et al., 1997). We stimulated primary cultured cells with DA or the D_1_R agonist SKF-38393 and observed that both treatments increased [^3^H] D-Aspartate release compared with the basal condition (Figure 1A). Because the DA-dependent modulation of D_1_R induces ascorbate release via the activation of the guanine exchange factor EPAC2 instead of PKA (da Encarnação et al., 2018), we evaluated the effect of EPAC2 activation in the release of [^3^H] D-Aspartate. Indeed, 8-pCPT-2′-O-Me-cAMP (Me-cAMP), a selective EPAC stimulator, also induced [^3^H] D-Aspartate release from retinal cells (Figure 1B), suggesting that the DA/D_1_R/EPAC2 signaling pathway can induce EAA release from retinal cultured cells.

**FIGURE 2:**
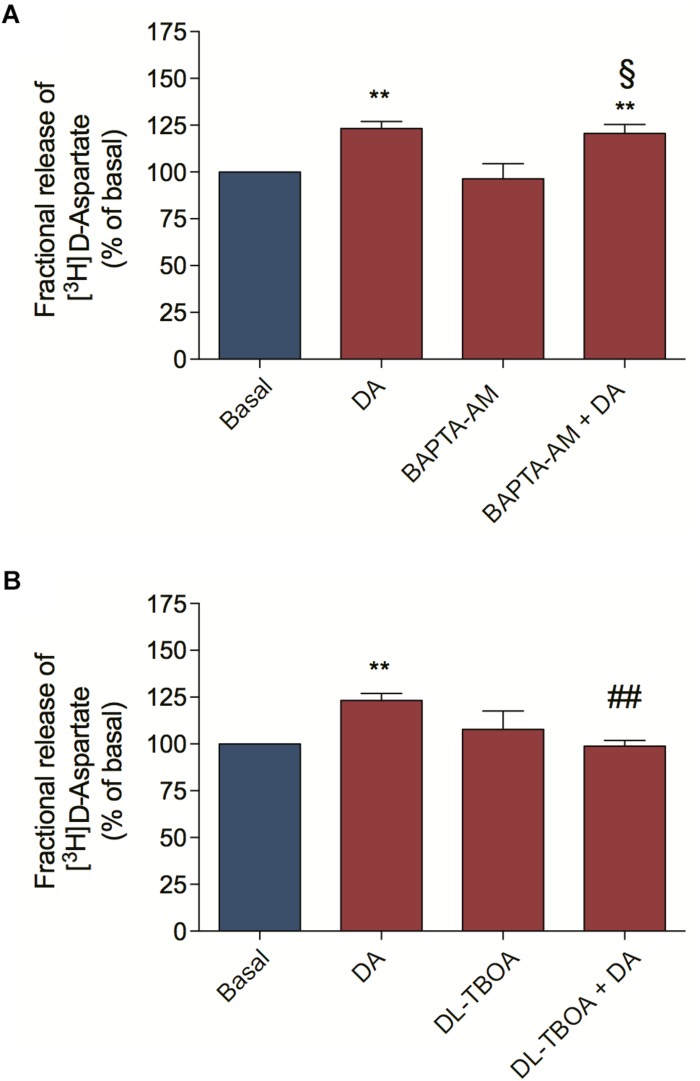
Dopamine-induced D-aspartate release is mediated by EAATs. Retinal cultures were incubated for 90 min with [^3^H] D-aspartate (1 μCi/mL), washed and processed for release experiments as described in section “Materials and Methods.” Cultures were pre-incubated with the intracellular Ca^2+^ chelator, BAPTA-AM (50 μM; **A**) or the selective non-transportable inhibitor of EAATs DL-TBOA (100 μM; **B**) for 10 min. Then, cultures were incubated with DA (50 μM; **A,B**) for an additional period of 10 min. The results represent the mean ± SEM of four independent experiments. Statistical analyses were performed using one-way ANOVA followed by the Bonferroni post-test. **p < 0.01 (relative to basal); ^#⁢#^*p* < 0.01 (relative to DA). ^§^ Not statistically different compared with DA. DA, Dopamine.

We also asked by which mechanism DA could mediate [^3^H] D-Aspartate release from retinal cells. [^3^H] D-Aspartate release in the retina can occur via calcium-dependent and calcium-independent mechanisms (Santos et al., 1996; de Freitas et al., 2016). In order to distinguish between them, we pre-incubated retinal cell cultures with BAPTA-AM to abolish cytosolic calcium mobilization and observed that BAPTA-AM treatment did not block the DA-induced [^3^H] D-Aspartate release (Figure 2A), concluding that DA induces [^3^H] D-Aspartate release through a mechanism independent of cytosolic calcium mobilization. Because reversion of EAATs is the main calcium-independent mechanism regulating the release of glutamate in the retina (de Freitas et al., 2016), we blocked EAATs-dependent transport with DL-TBOA and observed that incubation of retinal cells with DL-TBOA abrogated the DA-induced [^3^H] D-Aspartate release (Figure 2B). Altogether, these data suggest that DA, by activating a D_1_R/EPAC2 signaling pathway, induces EAA release from cultured retinal cells through an EAAT-dependent mechanism.

### EAA Released in Response to Dopamine Activates Ionotropic Glutamate Receptors Eliciting Ascorbate Release

**FIGURE 3:**
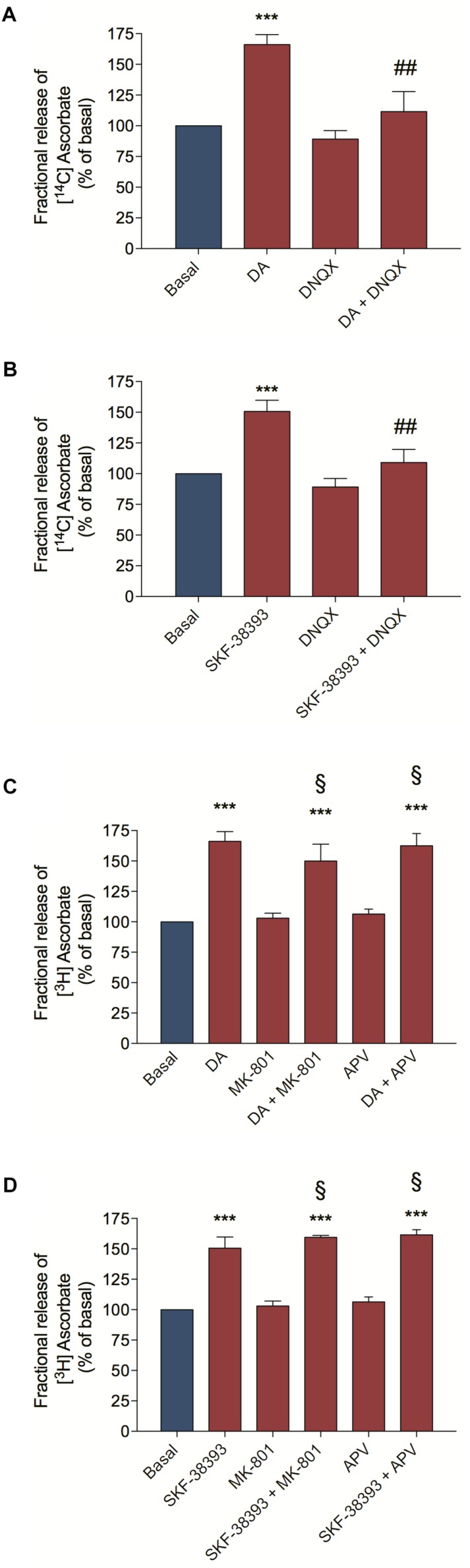
Dopamine-induced ascorbate release was inhibited by AMPA/Kainate receptors antagonist. Retinal cultures were incubated for 40 min with [^14^C] Ascorbate (0.3 μCi/mL), washed and processed for release experiments as described in section “Materials and Methods.” Cultures were pre-incubated with the AMPA/Kainate receptors antagonist DNQX (200 μM; **A,B**) or with the NMDA receptors antagonists, MK-801 (10 μM; **C,D**) or APV (100 μM; **C,D**) for 10 min. Then, cultures were incubated with DA (50 μM; **A,C**) or SKF-38393 (10 μM; **B,D**) for an additional period of 10 min. The results represent the mean ± SEM of three independent experiments. Statistical analyses were performed using one-way ANOVA followed by the Bonferroni post-test. ***p < 0.001 (relative to basal); ^#⁢#^*p* < 0.01 (relative to DA or SKF38393). ^§^ Not statistically different compared with DA. DA, Dopamine.

As we demonstrated above, DA is capable of inducing [^3^H] D-Aspartate release by the activation of D_1_R/EPAC2 signaling pathway. To demonstrate that the DA-induced EAA release and activation of glutamate ionotropic receptors is important for the DA-induced ascorbate release, we pre-incubated cultured retinal cells with DNQX, an AMPA/Kainate receptor antagonist and then stimulated cultures with DA or the D_1_R agonist SKF-38393. We observed that DNQX completely blocked the DA/D_1_R-induced ascorbate release (Figures 3A,B, respectively). Moreover, we also used NMDA receptor antagonists (MK-801 and APV) and analyzed if cooperation between AMPA/Kainate and NMDA receptors could control the DA/D_1_R-induced release of ascorbate. We observed that inhibiting NMDA receptors with MK-801 or with APV could not block the release of ascorbate from cultures stimulated with DA (Figure 3C) or with the D_1_R agonist SKF-38393 (Figure 3D). Overall, these data corroborate the hypothesis that DA induces EAA release followed by activation of AMPA/Kainate receptors to elicit ascorbate release from neuronal cells.

### DA-Induced Ascorbate Release Occurs via ERK MAP Kinases and PI3K/AKT Pathways

**FIGURE 4:**
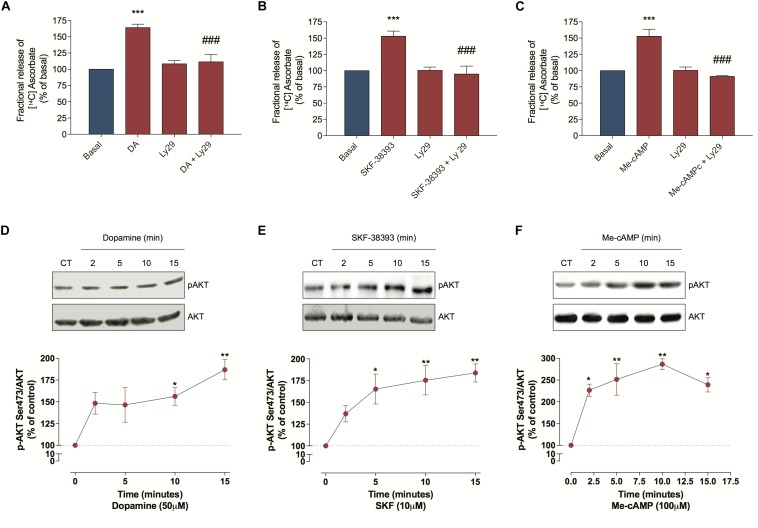
Dopamine-induced ascorbate release is mediated by EPAC/PI3K/AKT signaling pathway. **(A–C)** Cultures were incubated with [^14^C] ascorbate (0.3 μCi/mL) for 40 min, washed and processed for release experiments as described in section “Materials and Methods.” Cultures were pre-incubated with the PI3K blocker Ly294002 (5 μM) for 10 min. Then, cells were incubated with DA (50 μM; **A**), SKF-38393 (10 μM; **B**), or Me-cAMP (100 μM; **C**) either in presence or absence of Ly294002 (5 μM) for additional 10 min. The results represent the mean ± SEM of three independent experiments. **(D–F)** Cultures were incubated with DA (50 μM; **D**), SKF-38393 (10 μM; **E**), or Me-cAMP (100 μM; **F**) for different time periods (2; 5; 10, and 15 min) and then prepared for western blotting as described in section “Materials and Methods.” Data were quantified using ImageJ software and plotted as AKT^S473^/AKT ratio normalized to the control. DA time-curve (*n* = 3); SKF-38393 time-curve (*n* = 3) and Me-cAMP time curve (*n* = 3). The results represent the mean ± SEM. Statistical analyses were performed using one-way ANOVA followed by the Bonferroni **(A–C)** or Dunnet **(D–F)** post-test. *p < 0.05 (relative to control); **p < 0.01 (relative to control); ***p < 0.001 (relative to basal); ^#⁢#⁢#^*p* < 0.001 (relative to DA, SKF38393 or Me-cAMP). DA, Dopamine; Me-cAMP, 8-pCPT-2′-O-Me-cAMP; Ly29, Ly294002; SKF, SKF-38393.

As DA can induce some biological events via the PI3K/AKT pathway (Brami-Cherrier et al., 2002; Xu et al., 2013), we further evaluated PI3K involvement in the DA-induced ascorbate release. For this we treated cultured retinal cells with Ly294002, a PI3K inhibitor, and observed that this treatment blocked the DA, the SKF-38393 and the Me-cAMP-induced ascorbate release (Figures 4A–C), indicating that DA/D_1_R/EPAC promotes ascorbate release in a PI3K-dependent manner. To corroborate this hypothesis, we analyzed AKT phosphorylation at Ser473 and observed that DA, SKF-38393, or Me-cAMP were capable of increasing AKT^Ser473^ phosphorylation (Figures 4D–F). Therefore, these data suggest that DA-induced ascorbate release is mediated by EPAC/PI3K/AKT signaling pathway downstream of D_1_ receptors activation.

**FIGURE 5:**
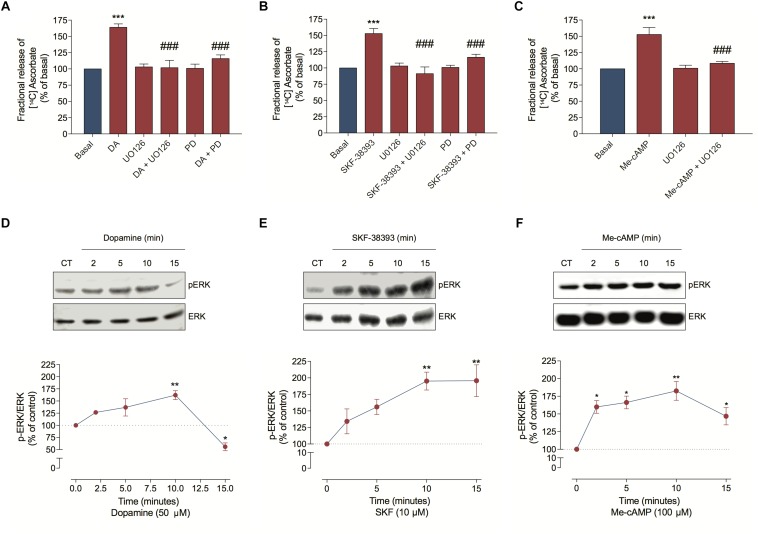
Dopamine-induced ascorbate release is mediated by EPAC/MEK/ERK signaling pathway. **(A–C)** Cultures were incubated with [^14^C] ascorbate (0.3 μCi/mL) for 40 min, washed and processed for release as described in section “Materials and Methods.” Cultures were pre-incubated with the MEK blockers UO126 (10 μM) or PD98059 (25 μM) for 10 min. Then, cells were incubated with DA (50 μM; **A**), SKF-38393 (10 μM; **B**) or Me-cAMP (100 μM; **C**) either in presence or absence of UO126 (10 μM) or PD98059 (25 μM) for an additional period of 10 min. The results represent the mean ± SEM of three independent experiments. **(D–F)** Cultures were incubated with DA (50 μM; **D**); SKF-38393 (10 μM; **E**) or Me-cAMP (100 μM; **F**) for different time periods (2; 5; 10, and 15 min) and then prepared for western blotting as described in section “Materials and Methods.” Data were quantified using ImageJ software and plotted as *p*-ERK/ERK ratio normalized to the control. DA time-curve (*n* = 3); SKF-38393 time-curve (*n* = 3) and Me-cAMP time curve (*n* = 3). The results represent the mean ± SEM. Statistical analyses were performed using one-way ANOVA followed by the Bonferroni **(A–C)** or Dunnet **(D–F)** post-test. *p < 0.05 (relative to control); **p < 0.01 (relative to control); ***p < 0.001 (relative to basal); ^#⁢#⁢#^ *p* < 0.001 (relative to DA, SKF38393, or Me-cAMP). DA, Dopamine; Me-cAMP, 8-pCPT-2′-O-Me-cAMP; PD, PD98059; SKF, SKF-38393.

Because DA can also stimulate ERK phosphorylation in neuronal cells (Xue et al., 2015), we investigated MEK/ERK involvement in ascorbate release. Hence, we inhibited this signaling pathway with UO126 or PD98059 and observed that both treatments completely blocked the DA, the SKF-38393 and the Me-cAMP-induced ascorbate release (Figures 5A–C). As expected, DA, SKF-38393, or Me-cAMP elicited ERK phosphorylation (Figures 5D–F), further demonstrating that the DA signaling increased MAP kinase activation. These data suggest that the DA-induced ascorbate release is mediated by DA/D_1_R/EPAC/MEK/ERK signaling pathway in cultured retinal cells.

### ERK MAP Kinases and PI3K/AKT Pathways Are Not Involved in the DA-Induced [^3^H] D-Aspartate Release

**FIGURE 6:**
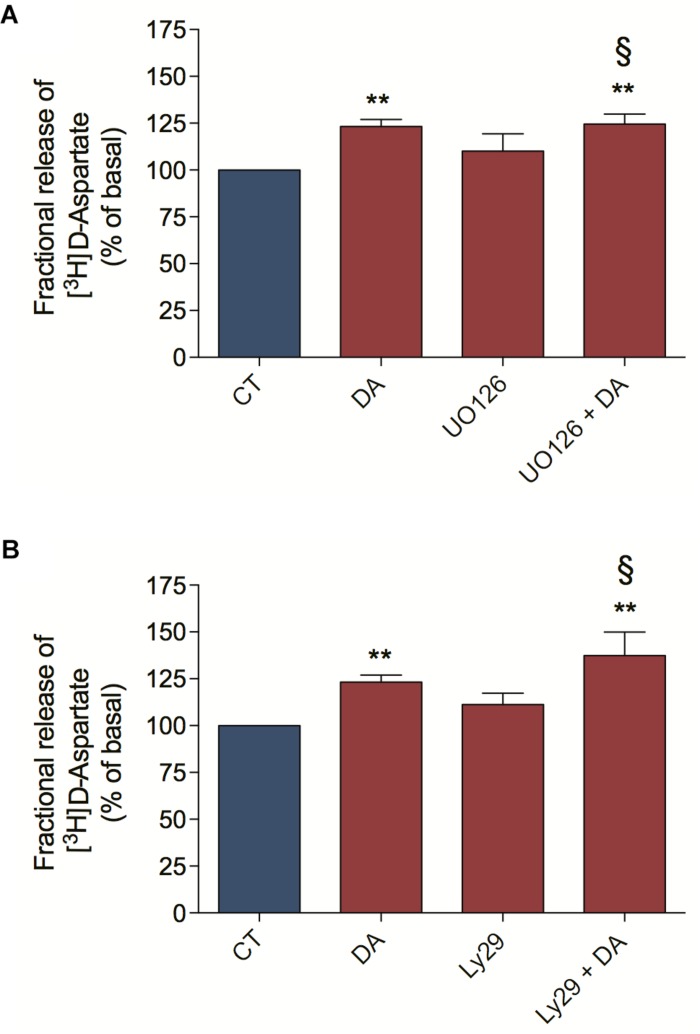
Dopamine-induced D-aspartate release is independent of ERK and AKT signaling pathways. Retinal cultures were incubated for 90 min with [^3^H] D-aspartate (1 μCi/mL), washed and processed for release experiments as described in section “Materials and Methods.” Cultures were pre-incubated with the MEK blocker UO126 (10 μM; **A**) or the PI3K blocker Ly294002 (5 μM; **B**) for 10 min. Then, cultures were incubated with DA (50 μM; **A,B**) for an additional period of 10 min. The results represent the mean ± SEM of four independent experiments. Statistical analyses were performed using one-way ANOVA followed by the Bonferroni post-test. **p < 0.01 (relative to basal). ^§^ Not statistically different compared with DA. DA, Dopamine; Ly29, Ly294002.

We characterize here that DA-induced ascorbate release is a mechanism dependent on EAA release, ERK MAP Kinases and PI3K/AKT signaling pathways. In order to better characterize the DA downstream signaling pathway involved in ascorbate release, we asked if the ERK MAP Kinases and the PI3K/AKT signaling pathways were upstream or downstream of the DA-induced [^3^H] D-Aspartate release. For that, we blocked the MEK/ERK signaling pathway with UO126 and observed that this treatment did not inhibit the DA-induced [^3^H] D-Aspartate release (Figure 6A). Also, we evaluated the PI3K/AKT signaling pathway by inhibiting PI3K with Ly294002 and observed that this treatment did not block the DA-induced [^3^H] D-Aspartate release (Figure 6B). Altogether, these data demonstrate that ERK MAP Kinases and PI3K/AKT signaling pathways are not involved in the DA-induced D-Aspartate release, positioning these signaling pathways downstream of EAA release.

## Discussion

**FIGURE 7:**
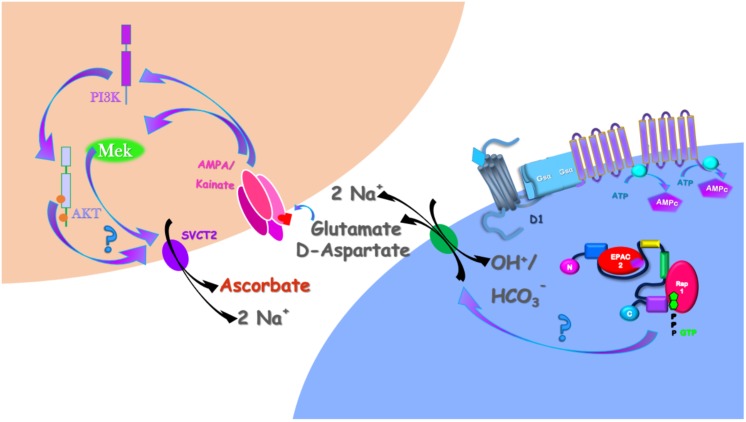
Schematic representation of the DA-induced ascorbate release signaling pathway in the retina. DA, by interacting with the D_1_R/cAMP/EPAC2 (da Encarnação et al., 2018) induces glutamate release by the EAAT reversion. At the extracellular milieu, glutamate interacts with AMPA/Kainate receptors that, in its turn, activates the MAP Kinases and the PI3K/AKT signaling pathways (Socodato et al., 2009, 2012; Mejia-Garcia et al., 2013). These signaling pathways, somehow, induces the SVCT2 reversion necessary to induce ascorbate release in the retina (Portugal et al., 2009; da Encarnação et al., 2018).

Here, we demonstrated that DA induces ascorbate release via a previous induction of [^3^H] D-Aspartate release (that can serve as proxy for the release of other EAA, including glutamate) through a calcium-independent and EAAT-dependent mechanism. Then, we revealed that DA-induced ascorbate release depended on downstream glutamate-mediated activation of ERK and AKT signaling pathways. Using an illustrative model (Figure 7), we concatenated these data with our previous published works. DA, acting through the D_1_R/cAMP/EPAC2 pathway

(da Encarnação et al., 2018), induces ascorbate release. Here, we demonstrated that DA/D_1_R/EPAC2 signaling pathway induces ascorbate release mediated by a previous release of EAA, here represented by D-Aspartate and Glutamate. As D-Aspartate could not activate AMPA/Kainate receptors (Kubrusly et al., 1998), we represented just the glutamate interacting in these receptors, which is in accordance with our previous work demonstrating that glutamate induces ascorbate release via activation of AMPA/Kainate receptors (Portugal et al., 2009). By interacting with AMPA/kainate receptors, glutamate stimulates ERK MAP Kinases (Socodato et al., 2009, 2012) and the PI3K/AKT (Mejia-Garcia et al., 2013) signaling pathways, which were involved in the DA-induced ascorbate release. Finally, as we previously reported (Portugal et al., 2009; da Encarnação et al., 2018), the mechanism eliciting ascorbate release is mediated by the reversion of SVCT2 (Figure 7).

The retinal cell culture paradigm we used is composed by neurons and Müller glial cells in a well-known proportion (Portugal et al., 2012). Moreover, DL-TBOA-sensitive glutamate transporters are expressed in both neurons and Müller glia in these cultures as well as D_1_Rs and EPAC2 (da Encarnação et al., 2018). Therefore, whether glutamate is being released from neurons, glial cells or both cells in response to DA needs further investigation. In any case, the ascorbate transporter SVCT2 is only present in neurons in retinal cultures (Portugal et al., 2009), thereby suggesting that ascorbate is released from neurons in response to DA/glutamate (Figure 7).

### Ascorbate Bioavailability and Neurodegenerative Disorders

Reduced ascorbate levels in the brain not only induce changes in mood, behavior, and motor performance (Brown, 2015) but also are directly involved in some neurological disorders such as Alzheimer’s and Huntington’s disease (Harrison and May, 2009; Harrison et al., 2009; Acuna et al., 2013; Dixit et al., 2015). Because SVCT2 is the main ascorbate transporter present in the brain, heterozygosity for a null allele for Slc23a2, the gene coding for SVCT2 in mice, leads to a decrease in 30% in their brain ascorbate levels (Sotiriou et al., 2002; Dixit et al., 2015). When SVCT2 heterozygous mice are intercrossed with a mouse model of Alzheimer’s disease, an aggravation of amyloid pathology and cognition impairment occurs (Dixit et al., 2015). Accordingly, ascorbate supplementation is capable of improving cognition in Alzheimer’s mice (Harrison et al., 2009). Moreover, abnormal ascorbate transport is observed in the R6/2 transgenic mouse model of Huntington’s disease (Dorner et al., 2009; Acuna et al., 2013). Reduction of ascorbate in the striatum is linked with the motor dysfunction observed in R6/2 Huntington’s disease mice (Dorner et al., 2009). Because the regulation of ascorbate transport could potentially mitigate some neurological hallmarks in these disorders (Harrison and May, 2009), a better understanding of the mechanisms regulating ascorbate transport and bioavailability could lead to improved therapeutic approaches to manage the neuronal damage observed in the abovementioned pathologies.

### Interaction Between DA and Glutamate Systems

Dopamine modulates glutamatergic signaling in different CNS regions (Scott et al., 2002; Tseng and O’Donnell, 2004; Fernandez et al., 2006; Surmeier et al., 2007), including the retina (Kolb, 2003). DA can alter the function of ionotropic glutamate receptors promoting exquisite control over neuronal and synaptic activity in different brain regions (Surmeier et al., 2007). However, much less is known about the control of EAA release by DA. For instance, DA acting via D_2_R inhibits the release of aspartate/glutamate in the retinal tissue and in striatal nerve terminals (Maura et al., 1989; Kamisaki et al., 1991) while here we demonstrate that DA acting via D_1_R/EPAC2 pathway increased the release of D-Aspartate in retinal cells. Interestingly, DA seems to be more efficient in inducing ascorbate than D-Aspartate release. As we demonstrated that aspartate/glutamate release is upstream of ascorbate release in retinal cultures, this difference could be explained by a signal amplification phenomenon, i.e., DA induces a small release of glutamate that, in turn, activates AMPA/Kainate receptors promoting a larger release of ascorbate.

### EAA Release From Retinal Cells

In general, the release of neurotransmitters in the CNS is mediated by exocytosis, a conserved mechanism for transmitter release across species. However, neurotransmitters and neuromodulators can also be released by the reversion of their transporters. In the retina, the transporter-mediated neurotransmitter release is a very common process for retinal physiology and has been described for classical neurotransmitters and neuromodulators, such as, GABA (Duarte et al., 1993; do Nascimento et al., 1998; Calaza et al., 2006), dopamine (Bugnon et al., 1995), adenosine (Paes-de-Carvalho et al., 2005) and the EAA, including D-Aspartate and glutamate (Duarte et al., 1996; Santos et al., 1996; de Freitas et al., 2016).

Excitatory amino acid release from nerve cells occurs through several distinct mechanisms that can be calcium-dependent and calcium-independent, including exocytosis and transporter-mediated processes. For instance, NMDA-evoked exocytic D-Aspartate release depends on extracellular calcium influx through the NMDA receptor channel, whereas AMPA-evoked exocytic D-Aspartate release depends on calcium influx through voltage-gated calcium channels (Santos et al., 1996). Moreover, D-Aspartate can be released simultaneously in a calcium-dependent and exocytic-independent manner (Bradford and Nadler, 2004). Here, we describe that the release of D-Aspartate, triggered by DA, was independent of calcium mobilization and required EAATs.

### Glutamate-Induced Ascorbate Release and the Involvement of Glutamate Ionotropic Receptors

Glutamate increases ascorbate release in cultured retinal cells (Portugal et al., 2009). Direct activation of NMDA receptors stimulates the release of ascorbate from neuronal cells (Portugal et al., 2009). However, the release of ascorbate induced by glutamate (Portugal et al., 2009) or by DA (data herein) was not blocked by NMDA receptor antagonists, such as MK-801 and APV. At least two hypothesis could explain the DA-mediated and glutamate-dependent stimulation of ascorbate release from neuronal cells: One hypothesis could be that activation of NMDA receptors promotes the release of glutamate from a glutamatergic neuron that, in turn, leads to activation of AMPA/Kainate receptors culminating in ascorbate release (Portugal et al., 2009). Another hypothesis could be that D_1_R and NMDA receptors are co-expressed in the same neuron in which D_1_R activation by DA leads to the activation of the cAMP/Csk/Src tyrosine kinase pathway decreasing the gating of NMDA receptors (Socodato et al., 2017). The first hypothesis is further corroborated by the fact that (1) the release of ascorbate elicited by glutamate (Portugal et al., 2009) or by DA (data herein) is abrogated by blocking AMPA/Kainate receptors with DNQX and (2) the NMDA-induced ascorbate release is also blocked by DNQX (Portugal et al., 2009). Although these hypotheses are not mutually exclusive, our data do suggest that the main driver for ascorbate release from neuronal cells is the activation of AMPA/Kainate receptors triggered by glutamate, which release was previously elicited by DA acting on D_1_R.

### ERK MAP Kinases and PI3K/AKT Pathways Involvement in the DA-Induced Ascorbate Release

Dopamine-induced ascorbate release was mediated by ERK MAP Kinases and the PI3K/AKT signaling pathways as UO126, a MAP inhibitor, and Ly294002, a PI3Kinhibitor, completely blocked the DA-induced ascorbate release. To corroborate this involvement, we also demonstrated that DA, SKF-38393 or Me-cAMP stimulates ERK and AKT phosphorylation. Also, we demonstrated that these signaling pathways are not involved in DA-induced D-aspartate release, suggesting that MAP Kinases and the PI3K/AKT signaling pathway are downstream of EAA release. Because glutamate promotes the phosphorylation of both ERK and AKT in retinal cells (Socodato et al., 2009, 2012; Mejia-Garcia et al., 2013), these signaling pathways might be downstream of AMPA/Kainate receptors stimulation. However, linking MAP Kinases and PI3K/AKT signaling pathways with the reversion of SVCT2 requires further investigation (Figure 7).

Overall, we showed that DA regulation of ascorbate release requires glutamate release and activation of AMPA/Kainate receptors. Mechanistically, DA activated D_1_R/EPAC2 to elicit an EAAT-dependent release of glutamate from retinal cells. Following DA-mediated glutamate release, glutamate promoted the activation of AMPA/Kainate receptors, increasing the activity of MAP Kinases and the PI3K/AKT, which coupled the reversion of SVCT2 to the release of ascorbate. Thus, our data suggest that a crosstalk between DA (the major catecholamine in the retina) and glutamate (the major excitatory neurotransmitter in the retina) controls the bioavailability of vitamin C in the retina.

## Author Contributions

CCP, TGE, and RPC conceived all the experiments. ID, ASR, and TGE performed [^3^H] Aspartate release experiments. CCP and TGE performed [^14^C] Ascorbate release experiments. CCP, TGE, RS, and NAO performed the western blots experiments. CCP, RS, ASR, and RPC wrote the manuscript. RPC provided the funding sources. All authors critically discussed the results and reviewed the final version of the manuscript.

## Conflict of Interest Statement

The authors declare that the research was conducted in the absence of any commercial or financial relationships that could be construed as a potential conflict of interest.
